# Early Interventions Following the Death of a Parent: Protocol of a Mixed Methods Systematic Review

**DOI:** 10.2196/resprot.7931

**Published:** 2017-06-29

**Authors:** Mariana Pereira, Iren Johnsen, May Aa Hauken, Pål Kristensen, Atle Dyregrov

**Affiliations:** ^1^ Center for Crisis Psychology Faculty of Psychology University of Bergen Bergen Norway; ^2^ Faculty of Psychology Department of Clinical Psychology University of Bergen Bergen Norway

**Keywords:** early intervention, parental loss, protocol, mixed methods, systematic review

## Abstract

**Background:**

Previous meta-analyses examined the effectiveness of interventions for bereaved children showing small to moderate effect sizes. However, no mixed methods systematic review was conducted on bereavement interventions following the loss of a parent focusing on the time since death in regard to the prevention of grief complications.

**Objective:**

The overall purpose of the review is to provide a rigorous synthesis of early intervention after parental death in childhood. Specifically, the aims are twofold: (1) to determine the rationales, contents, timeframes, and outcomes of early bereavement care interventions for children and/or their parents and (2) to assess the quality of current early intervention studies.

**Methods:**

Quantitative, qualitative, and mixed methods intervention studies that start intervention with parentally bereaved children (and/or their parents) up to 6 months postloss will be included in the review. The search strategy was based on the Population, Interventions, Comparator, Outcomes, and Study Designs (PICOS) approach, and it was devised together with a university librarian. The literature searches will be carried out in the Medical Literature Analysis and Retrieval System Online (MEDLINE), PsycINFO, Excerpta Medica Database (EMBASE), and Cumulative Index to Nursing and Allied Health Literature (CINAHL). The Mixed Methods Appraisal Tool will be used to appraise the quality of eligible studies. All data will be narratively synthetized following the Guidance on the Conduct of Narrative Synthesis in Systematic Reviews.

**Results:**

The systematic review is ongoing and the data search has started. The review is expected to be completed by the end of 2017. Findings will be submitted to leading journals for publication.

**Conclusions:**

In accordance with the current diagnostic criteria for prolonged grief as well as the users’ perspectives literature, this systematic review outlines a possible sensitive period for early intervention following the death of a parent. The hereby presented protocol ensures the groundwork and transparency for the process of conducting the systematic review.

**Trial Registration:**

International Prospective Register of Systematic Reviews (PROSPERO) CRD42017064077; http://www.crd.york.ac.uk/PROSPERO/display_record.asp?ID=CRD42017064077 (Archived by WebCite at http://www.webcitation.org/6rMq6F0fv)

## Introduction

### Background

This paper presents the study protocol of a mixed methods systematic review on early bereavement interventions following the loss of a parent, adhering to the Preferred Reporting Items for Systematic Review and Meta-Analysis Protocols guidelines (PRISMA-P) [1]. This protocol paper seeks to strengthen the quality, reliability, and transparency all the way through the completion of the systematic review. 

The death of a parent has been documented as a major stressful and disturbing experience for children [2,3]. Parentally bereaved children are more prone to functional impairment and other multiple negative outcomes, including psychological and behavioral problems [4,5]. Although former clinical assumptions stressed children’s lack of ability to grieve [6], it is now well accepted that bereaved children experience a grieving process [7] and some may develop extended psychiatric conditions [8-11]. Moreover, the death of a parent is also associated with an increased mortality risk in children [12,13]. As a result, a diversity of theoretical frameworks and psychosocial interventions have been proposed.

Grief intervention usually consists of quite diverse intervention approaches such as, for example, self-help, family interventions, support groups, counseling, and therapy. Such interventions are delivered by a variety of (para)professionals (eg, psychologists, social workers, nurses, pastoral staff) in varied formats (eg, individual or group, Internet, telephone, home visiting) [14]. Similarly to what was shown in the literature for bereaved adults [15], quantitative reviews on the effectiveness of bereavement interventions for children showed small to moderate effect sizes [16,17]. Nonetheless, interventions targeting high-risk children [16,17] and interventions starting closer to the loss were proved to be more effective [16]. On average, the length of time to initiate intervention after the death was a year and a half, but several studies presented a time interval of 5 years postloss.

An emphasis has been placed in starting interventions early in the mourning process [18,19]. When interventions take place later in the mourning trajectory, children may not be focused on their loss any longer and may have changed in a maladaptive way [16]. Therefore, early psychological interventions for bereaved children and their parents have been highlighted as a tool to decrease acute distress levels [20] and prevent future psychopathology [11,21], namely posttraumatic and complicated grief reactions [22]. In addition, bereaved parents require early qualified help not just for themselves but for their children in particular [23,24]. Likewise, bereaved children ask for early notification and involvement [25,26]. Nevertheless, contradicting results concerning grief therapy for adults still prevail. While some documented that interventions closer in time to the death are more effective [18], others did not find a significant relation between the effect of time since loss on outcome [15]. Additionally, a meta-analysis examining the short- and long-term effects of grief interventions for adults did not support the usefulness of preventive approaches [19]. The authors pointed out a series of methodological limitations among preventive studies such as, for instance, the lack of proper grief measures in screening at-risk groups. However, they did not elaborate on the fact that some studies did not report the mean time since death at study entry, whereas other studies reported a broad time range of 1 month to 2 years postloss. Such diverse timeframes have been noticed as one of the accountable variables for lowering the main effects of grief interventions [16,27].

According to the *International Classification of Diseases*, 11^th^ Revision (ICD-11) and the *Diagnostic and Statistical Manual of Mental Disorders*, 5^th^ Edition (DSM-5), it looks like the long time intervals of the contemporary quantitative reviews run counter to the viewpoint emphasizing early intervention. The proposal for ICD-11 revision acknowledges grief reactions as a possible form of psychiatric disorder whenever severe grief complications persist beyond 6 months postloss [28]. The current DSM-5 revision suggests a minimal watchful period of 6 months postloss for children and 12 months for adults [29]. A previous review suggested that grief counseling would be more successful if provided within 6 to 18 months following the death. However, this same review also considered that different types of support may be required early and/or later in the bereavement process [27]. Despite the fact that the focus on early intervention has been raised by both clinicians and researchers, there is a profound lack of unanimity. Its conceptualization, timeframes, and effectiveness have not been thoroughly explored. The dearth of high-quality and methodologically equivalent studies [30-32] seem to have led to the general conclusion that the grief field remains in its infancy [33].

Building on the existing quantitative reviews for bereaved children, evidence from qualitative, quantitative, and mixed methods studies will be collected. Based on the ICD-11 and DSM-5 diagnosing systems and the users’ perspectives studies, the mixed methods systematic review will focus on intervention studies initiated within 6 months after parental death in regard to the prevention of grief complications. The bereavement interventions will be dependent on the child's age (≤18 years of age), a setting that is compatible with the early intervention standpoint of promoting an optimal developmental trajectory.

### Objectives

The overall purpose of the mixed methods systematic review is to provide a rigorous synthesis of early intervention after parental death in childhood. The following two questions will be addressed:

1. What are the rationales, contents, timeframes, and outcomes of early bereavement care interventions for children and/or their parents?
2. What is the quality of current early bereavement care intervention studies for children and/or their parents?

## Methods

The Preferred Reporting Items for Systematic Review and Meta-Analysis (PRISMA) [34] will guide the completion and reporting of the systematic review. The Covidence online systematic review platform (www.covidence.org) will be used to support the screening, selection, and data extraction stages.

### Eligibility Criteria

As shown in Textbox 1, the Population, Interventions, Comparator, Outcomes, and Study Designs (PICOS) structured approach [34] is used to frame inclusion and exclusion criteria for studies. In view of the overall family context, each study will contemplate parentally bereaved children (≤18 years of age) and/or their parents with no limits on further sociodemographic indicators such as gender, ethnicity, or socioeconomic status. Any causes of parental death will be included, whether due to illness, accidents, or other causes. Any type of bereavement psychosocial intervention (eg, crisis intervention, support groups, counseling) starting up to 6 months postloss will be included.

The review will encompass all types of study designs (quantitative, qualitative, and mixed methods). It will include studies published in English language peer-reviewed scientific journals as well as dissertations. Exclusions will apply to systematic or other forms of literature reviews, letters, commentaries/editorials, and conference abstracts/presentations. Study protocols will only be consulted to provide deficient details of later related articles.

**Study eligibility criteria.**

PICOS framework:

Population

- Inclusion—parentally bereaved children and/or their parents
- Exclusion—children aged >18 years

Interventions

- Inclusion—any type of bereavement intervention starting within the first 6 months postloss
- Exclusion—any type of bereavement intervention starting after 6 months postloss

Comparator

Not applicable in the context of this mixed methods systematic review

Outcomes

Not applicable in the context of this mixed methods systematic review

Study design(s)

- Inclusion—peer-reviewed quantitative, qualitative, and mixed methods studies and dissertations
- Exclusion—systematic or other forms of literature reviews, stand-alone study protocols, letters, commentaries/editorials, and conference abstracts/presentations

### Screening and Selection Process

Two reviewers (MP and IJ) will independently undertake the database searches. An initial-stage screening of titles and abstracts will be performed, and the studies will be assessed against the predetermined inclusion and exclusion criteria. Articles that explicitly do not meet the eligibility criteria will be excluded, while potentially eligible studies will be imported into EndNote citation software (Clarivate Analytics) and duplicates will be deleted. The full text of potentially eligible articles and studies for which a decision grounded on title/abstract cannot be made will be saved for investigation. Any disagreements will be reconciled through discussion and achieving consensus. A second-stage screening of the full-text articles will be independently conducted by MP and IJ for further eligibility assessment. Reasons for exclusion will be documented for future inclusion in the PRISMA flow diagram [34]. Following this, a citation search of all eligible studies and any pertinent reviews attained during the first- and second-stage screenings will be performed to search for additional studies. Once again, disagreements will be resolved via discussion. Multiple articles pertaining to the same study will be gathered and placed under the main study.

### Data Sources and Search Strategy

A comprehensive literature search will be carried out on the largest medical, psychological, and nursing databases: Medical Literature Analysis and Retrieval System Online (MEDLINE) (via Ovid), PsycINFO (via Ovid), Excerpta Medica Database (EMBASE) (via Ovid), and Cumulative Index to Nursing and Allied Health Literature (CINAHL) (via EBSCO). To secure a contemporary overview, possible eligible studies will be searched from January 1980.

The search strategy was developed using the PICOS framework [34]. It was informed by the methods sections of previous reviews on bereavement interventions [32,35] and was devised together with a university librarian. The search terms will combine keywords referring to the intervention and the population. Table 1 shows an example of the MEDLINE search strategy that will also be used for the other databases. There will be no methodological filters so that quantitative, qualitative, and mixed methods studies can be screened.

**Table 1:** MEDLINE search strategy.

Number	Search terms
1	interven*.ti,ab,id.
2	program*.ti,ab,id.
3	counsel*.ti,ab,id.
4	support*.ti,ab,id.
5	prevent*.ti,ab,id.
6	1 or 2 or 3 or 4 or 5
7	exp “Early Intervention (Education)”/ or exp Crisis Intervention/
8	6 or 7
9	death.ti,ab,id.
10	dying.ti,ab,id.
11	grie*.ti,ab,id.
12	los*.ti,ab,id.
13	9 or 10 or 11 or 12
14	parent*.ti,ab,id.
15	father*.ti,ab,id.
16	mother*.ti,ab,id.
17	care-giver*.ti,ab,id.
18	caregiver*.ti,ab,id.
19	14 or 15 or 16 or 17 or 18
20	13 ADJ3 19
21	exp Parental Death/
22	20 or 21
23	8 and 22

### Data Extraction

A data extraction template was developed using Cochrane existing guidelines [36] as well as current proposals identifying central aspects of interventions [37]. It comprises information concerning (1) eligibility (eg, child’s age, time since death, reasons for exclusion); (2) study characteristics (eg, aims, design, causes of death); (3) participant demographics (eg, age, sex, socioeconomic status); (4) intervention features (eg, theoretical basis, setting, contents/components); and (5) outcomes (eg, frameworks, types of measurements). This data extraction form was independently piloted by two reviewers (MP and IJ).

Despite the likelihood of some unreported data, specifically regarding intervention features, this review will not ask original authors to provide additional information. This is in line with the overall aim to specify and qualify what has been disclosed in peer-reviewed publications. Two reviewers (MP and IJ) will independently extract the data and disagreements will be reconciled through discussion.

### Quality Assessment

The interest in mixed methods studies has been increasing over the last 2 decades. However, the emergence of mixed methods systematic reviews is rather novel and there is no consensus regarding its best critical appraisal measures [38]. The Mixed Methods Appraisal Tool (MMAT) [39] consists of a pilot-tested quality assessment instrument that simultaneously assesses the most prevalent types of quantitative, qualitative, and mixed methods studies. The MMAT was chosen because it allows different study designs to be qualified with the same measure. It was specifically developed to be used in mixed methods systematic reviews [38], and it has been used by others in the grief field [35].

Two reviewers (MP and MH) will independently use the MMAT to assess the quality of eligible studies. Both quality scores and general brief descriptions will be provided, and the results will be compared for consistency. Disagreements will be resolved by discussion.

### Data Synthesis

Since this review covers a wide range of research designs and multiple findings, it will make use of an interpretative framework. This approach is well suited to systematic reviews including studies expected to be too heterogeneous to ensure a quantitative overview [40]. The Guidance on the Conduct of Narrative Synthesis in Systematic Reviews, proposed by Popay and colleagues [40], will be followed. It seeks to “tell the story” of the review findings through the iterative implementation of four stages: (1) developing a theory of how the intervention works, why, and for whom; (2) developing a preliminary synthesis of findings of included studies; (3) exploring relationships in the data; and (4) assessing the robustness of the synthesis. In line with these guidelines, a number of different tools and techniques (eg, textual descriptions, tabulation, grouping and clusters, qualitative case descriptions, and reflecting critically on the synthesis) will be used to accommodate the data at each stage. This will be done through an iterative and inductive process, and the emerging results will be discussed with the research team throughout.

## Results

This systematic review is in progress. The data search has started and the review is planned to be completed by winter 2017. The results will be submitted to leading journals for publication.

## Discussion

The systematic review planned in this protocol paper will convey an up-to-date picture of early intervention after parental death in childhood. The bereavement interventions will be dependent on the child's age (≤18 years of age). This setting is very important to the early intervention field, which seeks to prevent initial dysfunctional manifestations and promote a more adaptive developmental pathway [22]. Although previous meta-analyses were carried out to inspect the effectiveness of interventions for bereaved children [16,17], none has focused exclusively on the loss of a parent or the length of time since death as regard to the prevention of grief complications. The present protocol delimits the timing to start intervention within 6 months postloss, thus it may convey a pertinent boundary for early bereavement care. The rationale for this period is grounded in the current ICD-11 and DSM-5 diagnostic criteria, as well as the literature granting voice to the bereaved families’ needs. Another important feature is the inclusion of qualitative, quantitative, and mixed methods designs. Mixed methods reviews, and their reliance on narrative synthesis, broaden the interventions’ background and the explanations for their impact, resulting in more thorough implications for future research, policy, and practice [41,42]. Additional strengths are the use of contemporary recommendations for describing interventions [37]. These guidelines were slightly adjusted to the bereavement intervention field and added to our data extraction template. A precise and complete description of the interventions is an essential condition for comparing studies, relating intervention components and outcomes, testing theory [37], and transferring results into clinical practice [43].

While researchers have been calling for more homogeneous and methodologically sound studies [30-32], bereavement care services have developed practices based on experience and feedback from the users of the services. This mixed methods systematic review has the potential to shed light on much of the uncertainty around the conceptualization and helpfulness of preventive bereavement interventions for children who lose a parent to death. It will provide a rigorous synthesis of the rationales, contents, timeframes, and outcomes of early bereavement care interventions following the death of a parent in order to compile evidence about the results and quality of current intervention studies. These findings will convey an important input to recommendations of good practice concerning both research and public mental health care.

## Abbreviations

CINAHL: Cumulative Index to Nursing and Allied Health Literature
DSM-5: Diagnostic and Statistical Manual of Mental Disorders, 5thEdition
EMBASE: Excerpta Medica Database
ICD-11: International Classification of Diseases, 11thRevision
MEDLINE: Medical Literature Analysis and Retrieval System Online
MMAT: Mixed Methods Appraisal Tool
PICOS: Population, Interventions, Comparator, Outcomes, and Study Designs
PRISMA: Preferred Reporting Items for Systematic Review and Meta-Analysis
PRISMA-P: Preferred Reporting Items for Systematic Review and Meta-Analysis Protocols
